# Matters of life and death: the role of chromatin remodeling proteins in retinal neuron survival

**DOI:** 10.1007/s12177-012-9080-3

**Published:** 2012-03-17

**Authors:** Pamela S. Lagali, David J. Picketts

**Affiliations:** 1 Regenerative Medicine Program, Ottawa Hospital Research Institute, 501 Smyth Road, Ottawa, ON Canada K1H 8L6; 2 Department of Biochemistry, Microbiology and Immunology, University of Ottawa, 451 Smyth Road, Ottawa, ON Canada K1H 8M5

**Keywords:** Chromatin remodeling, Neuronal survival, Retina, Neurodegeneration, Epigenetics, Retinal degeneration

## Abstract

Retinal neurons are highly vulnerable to a diverse array of neurotoxic stimuli that leads to their degeneration, which is a major contributor to blindness. This review summarizes the role of epigenetic factors in mediating neuronal homeostasis and survival to protect against cell death and neurodegenerative conditions. Studies in human patients and mouse models implicate numerous chromatin modifications in neuroprotective processes including histone protein acetylation and methylation, DNA methylation, and ATP-dependent nucleosome remodeling. Recent research has begun to uncover specific epigenetic mechanisms invoked by neurotoxic stimuli. Continued investigation in this area will be the key to the generation of therapeutic strategies for the intervention of retinal neurodegenerative diseases.

## Introduction

The development of the nervous system from primordial structures occurs through a highly organized and intricately regulated series of events involving cell proliferation, differentiation, migration and maturation, culminating in the establishment of numerous neuronal cell types that participate in complex neural circuits and networks. Once fully differentiated, neurons are required to carry out their functions for the lifespan of the organism as most are considered irreplaceable if lost. While adult neurogenesis does occur in select regions of the central nervous system (CNS), the vast majority of neuronal cell types cannot be regenerated naturally from existing cellular pools. This implies that neurons are required to remain intact and functional in order for proper neuronal communication to persist and to avoid deficits in neural activity that may occur through accumulated losses over time. In order to overcome the wide range of insults and stressors encountered over a lifetime, neurons must be able to rapidly react to changing conditions and appropriately modify gene expression to maintain cellular integrity and functionality.

To quickly respond to extracellular cues and signals, the cell relies on epigenetic mechanisms of transcriptional regulation. Changes in gene expression occur as a result of alterations in the structure of chromatin that either allow or restrict access to the regulatory regions that control transcriptional activity. The basic subunit of chromatin is the nucleosome, consisting of 146 base pairs of DNA wrapped around an octamer of two copies of each of the core histone proteins H2A, H2B, H3, and H4 [1]. The linker histone H1 binds to the DNA between nucleosomes to enable further chromatin compaction. It is through the dynamic reorganization of nucleosomes and higher-order chromatin structure that genetic regulatory elements are made accessible to transcription factors to modify the expression of specific genes [2–4]. Covalent modifications of DNA and histone proteins disrupt chromatin compaction and also serve as landmarks that are recognized by chromatin-binding proteins that initiate downstream genetic processes. The enzymes that catalyze DNA and histone modification as well as the factors that interact with these marks are collectively termed chromatin remodeling proteins. The finding that mutations in a growing number of these proteins cause developmental and neurodegenerative defects is evidence of their critical function in the CNS from the embryo right through adulthood.

The development of the retina follows defined genetic programs that have been extensively characterized [5, 6]. An increasing number of studies describe interactions between the transcription factors that control the development of the retina and various chromatin remodeling proteins that may contribute to the execution of critical developmental programs [7]. However, much less attention has focussed on the epigenetic regulation of retinal neuron maintenance and homeostasis. This review describes the role of chromatin remodeling proteins in mediating survival or loss of neuronal cells in the retina under various conditions, including during developmental stages, in response to neurotoxic stimuli, and in heritable retinal degenerations. Neuroprotective mechanisms and strategies that follow from these findings may enable the development of novel therapeutics for treating human retinal degenerative conditions.

## Histone modifications impact retinal neuron survival

Histone proteins can be post-translationally modified by a variety of enzyme activities, resulting in their acetylation, methylation, phosphorylation, sumoylation, ubiquitination, ADP-ribosylation, deimination, and β-N-acetylglucosamination [8]. These covalent modifications can alter the association of histones with DNA and can also serve as a scaffold for the recognition and binding of other proteins that ultimately affect chromatin compaction and transcriptional regulation [9, 10]. Histone acetylation and methylation and the enzymatic activities that create or remove these marks have been implicated in the survival of CNS neurons.

### Histone acetylation

The transfer of an acetyl group to the lysine side chains of histone proteins causes the neutralization of the positive charge on the lysine residues and can therefore disrupt the interactions between histones and DNA within nucleosomes. Acetylation has been described for all of the core histone proteins and is generally associated with chromatin relaxation and transcriptional activation, while deacetylation favors chromatin condensation and transcriptional repression. The extent of post-translational acetylation is determined by the enzymatic activities of histone acetyltransferases (HATs) and histone deacetylases (HDACs). It is the net effect of both HAT and HDAC activities that determines the overall acetylation level of histones and impacts the transcriptional status of particular genes.

The acetylation of histones has been the most extensively studied chromatin modification in the context of neurodegenerative processes. Despite the different pathophysiologies associated with various neurodegenerative diseases, in vivo models demonstrate that a common feature of the degenerative process in the affected neurons is a reduction in the global levels of histone acetylation [11–13]. Consistent with this observation is the association of reduced or absent acetyltransferase activity mediated by CREB-binding protein (CBP)/p300 in these models [11, 12]. In the retina, the CBP and p300 HATs can form complexes with a variety of transcription factors that play critical roles in gene expression programs that direct retinal cell differentiation and maturation, including Crx, Pax6, Prox1, Mash1, NeuroD1, Sox9, RXRγ, and pRb [7]. Transcriptional activation of target genes by these factors can in turn be facilitated through the CBP/p300-mediated acetylation of histones to create permissive chromatin configurations that promote gene expression. Indeed, such is the case for the homeodomain transcription factor Crx, whose inactivation results in a retinal degenerative phenotype [14]. Moreover, mutations in Crx target genes also cause retinal degeneration [15]. Crx binds to the regulatory regions of its target photoreceptor genes, where it recruits HATs that acetylate histone H3, to enable chromatin remodeling and subsequent binding of transcriptional coactivators and RNA polymerase II for transcription to occur [16]. Since loss of Crx leads to retinopathy, it may follow that loss of the HAT activity and H3 acetylation on its target gene promoters may also result in photoreceptor degeneration. In *Crx* knockout mice, binding of HATs to target promoters and the levels of acetylated histone H3 on these promoters was reduced [16], and expression of the target genes was concomitantly reduced [14].

Similar to CBP and p300, the GCN5-HAT component of the multi-subunit SPT3-TAF9-ADA-GCN5 acetyltransferase (STAGA) transcription coactivator complex can interact with Crx and is associated with histone H3 acetylation on its target gene promoters [16]. The interaction of GCN5 and Crx is mediated by the ataxin-7 protein, another component of the STAGA complex [17]. Mutation of *ataxin-7* results in inhibition of HAT function, reduced H3 acetylation and reduced Crx occupancy of target genes [17], and causes neurodegeneration of the retina and brain [18, 19]. In addition to altered transcription profiles in photoreceptor cells, *ataxin-7* mutant mice also exhibit dramatic reorganization of chromatin correlated with reduced expression and abnormal distribution of the linker histone H1c in rod photoreceptor nuclei; however, global histone acetylation levels are unchanged [20]. It is possible that aberrant ataxin-7 function leads to altered targeting of HAT complexes, causing inappropriate acetylation and activation of otherwise repressed genes, and contributing to the upregulation of some photoreceptor genes that are observed in these mice [21]. Nevertheless, it appears that the survival of retinal neurons is impacted by multiple mechanisms involving changes in histone processing and dynamics.

This is further supported by the important role of HDACs that has been demonstrated in various mouse models of retinal neurodegeneration. In the *rd1* mouse model of retinitis pigmentosa, reduced histone acetylation levels were detected in photoreceptors but not other retinal cell types that do not undergo degeneration [22]. This was related to elevated HDAC activity predominantly attributed to class I and II HDACs. Furthermore, protein hypoacetylation and increased HDAC activity occurred in *rd1* photoreceptor cells undergoing apoptosis which could be protected from cell death by exposure to HDAC I/II inhibitors [22]. HDAC inhibition was also shown to promote retinal ganglion cell survival in optic nerve crush-induced neurodegeneration [23]. However, inhibition of class I/II HDACs can lead to broadly distributed apoptotic cell death in wild-type retinas [24], indicating that disruption of normal levels of protein acetylation can be cytotoxic, while reduction of HDAC overactivity occurring in retinal degeneration may result in the normalization of pathophysiological acetylation levels and subsequent neuroprotection. Additional studies in which resting levels of histone acetylation in neuronal cells under normal conditions are altered by either exposure to HDAC inhibitors [25, 26] or elevation of HATs [27, 28] further suggest that hyperacetylation of histones is toxic for neurons and that disturbing the sensitive balance between HAT and HDAC activities in either direction can trigger neuronal cell death.

Class III HDACs are also involved in mediating neuronal survival. A neuroprotective role for the Sirt1 histone deacetylase has been demonstrated in various neurodegenerative disease conditions [29–32]. Consistent with this, Sirt1 protein distribution is altered in degenerating retinas of *rd10* mice, where it co-localizes with apoptotic photoreceptors as well as pro-apoptotic proteins in the outer nuclear layer of the retina at the peak of cell death, and after which, its retinal expression is dramatically reduced [33]. It is hypothesized that the neuroprotective effects of Sirt1 are lost in the *rd10* photoreceptors due to its cellular mislocalization and reduced level of expression, therefore resulting in the degeneration of these cells.

Individual HDACs also appear to function in distinct neuronal survival pathways. Specific inactivation of HDAC1 in post-mitotic primary neurons results in significant cell death, while increased HDAC1 activity is protective against neurotoxicity in vivo [34]. HDAC4 has a neuroprotective role in the retina, as overexpression causes reduced levels of naturally occurring bipolar cell death during development and also rescues rod and cone photoreceptor cell loss in *rd1* mice [35]. Accordingly, inhibition of HDAC4 function in wild-type retinas by RNA interference induces significant cell loss due to apoptosis, indicating that HDAC4 is required for retinal neuron survival [35]. In contrast, HDAC5 or HDAC6 was unable to mediate rescue of the photoreceptors in the *rd1* mice, despite the ability of HDAC6 to rescue neurodegeneration in a *Drosophila* model of spinobulbar muscular atrophy [36]. In optic nerve injury-induced retinal neurodegeneration, apoptotic retinal ganglion cells (RGC) exhibit increased HDAC activity, reduced levels of acetylated histone H4, and downregulation of RGC marker and survival genes [37]. Only HDAC3 was shown to translocate to the nuclei of apoptotic RGC and exhibited increased and sustained expression that was consistent with the time course of acetylated H4 reduction following optic nerve crush in these mice [37]. This series of studies demonstrates that the use of broad HDAC inhibitors as potential therapeutic agents for the impaired histone acetylation levels and/or reduced HAT activities associated with transcriptional dysregulation and neuronal death must take into account the roles of specific HDACs in mediating retinal cell survival in physiological and pathological contexts.

### Histone methylation

Histone methylation is a non-charge neutralizing modification that occurs on specific lysine and arginine residues of histones H3 and H4. Methylation of particular lysine side chains can act as either permissive (e.g., H3K4, H3K36, and H3K79) or repressive (e.g., H3K9, H3K27, and H4K20) marks in the context of transcription. However, the extent of methylation of these residues can also affect their function in transcriptional activation or repression, with differential effects mediated by mono-, di- or trimethylation, thereby adding another layer of complexity [38, 39].

Similar to histone acetylation, histone methylation appears to be involved in the maintenance of different retinal cell types. The Purkinje cell degeneration (*pcd)* mouse is characterized by postnatal loss of several neuronal cell types including retinal photoreceptors [40, 41]. Neurodegeneration in these mice is associated with profound chromatin disorganization, an increase in the levels of trimethylated histone H4 at lysine 20, and transcriptional silencing [42]. While only Purkinje cells were examined in this study, the mechanisms underlying cell death may be relevant to the rods and cones of the retina that are also lost in these mice.

Specific methyltransferase activities are important for retinal ganglion cell survival, as inhibitors of the Ezh2 and G9a histone methyltransferases can induce apoptosis of these neurons in vitro [43]. Other methyltransferases have also been implicated in neurodegenerative processes. The association of the Suv39H1 histone methyltransferase with p130, the Rb protein family member that is required for the survival of neurons in vitro, is lost in response to apoptotic stimuli, and this is accompanied by a substantial reduction in histone H3 methyltransferase activity [44]. Furthermore, expression of a p130 mutant that fails to interact with Suv39H1, or mutant Suv39H1 that lacks histone methyltransferase activity, leads to death of cultured neuronal cells [44]. Suv39H1 functions as a chromatin-modifying transcriptional silencer and, in this study, was found to cause derepression of pro-apoptotic genes when its interaction with p130 on the promoters of these genes was lost. The mutation underlying adult-onset cerebellar Purkinje cell degeneration occurring in the robotic mouse mutant lies within the gene that encodes Af4, a component of a chromatin remodeling complex which contains the DOT1 histone H3 lysine 79 methyltransferase [45]. The *Af4* mutation causes a gain-of-function resulting in protein stabilization and accumulation, and increased levels of H3K79 methylation were correspondingly detected in cerebellar homogenates, which in turn is presumed to lead to transcriptional dysregulation in Purkinje neurons [46]. Therefore, the specific methylation of particular histones, as catalyzed by specific enzymes, is important for directing pro-survival gene expression in the retina as well as in other regions of the CNS. These studies also demonstrate that both elevated and reduced levels of histone methylation can contribute to altered transcription that leads to neuronal cell death, further underscoring the importance of tight regulation of histone post-translational modification.

## DNA methylation defects lead to neurodegeneration

DNA methylation is generally associated with transcriptional repression, either through direct physical inhibition of transcription factor binding to methylated promoters, or by the association of methyl-CpG-binding domain (MBD) protein-containing repressive complexes with gene promoters [47]. Two DNA methyltransferases (Dnmts), Dnmt3a and Dnmt3b, function to establish de novo DNA methylation patterns during mammalian embryonic development, while a third enzyme, Dnmt1, acts as a maintenance methyltransferase and preferentially methylates hemi-methylated DNA generated following DNA replication [48].

A number of studies involving CNS-specific knockouts of Dnmts implicate altered DNA methylation in neurodegenerative phenotypes. Conditional deletion of *Dnmt1* in mouse CNS precursor cells in vivo leads to global DNA hypomethylation and postnatal death of neurons in multiple brain regions [49]. *Dnmt1* deletion targeted to the dorsal forebrain causes severe and progressive degeneration of cortical and hippocampal neurons due to hypomethylation-induced apoptotic cell death occurring both pre- and postnatally [50]. This was accompanied by electrophysiological deficits, neurobehavioural defects in learning and memory, and deregulation of neuronal gene expression [50, 51]. These studies demonstrate the importance of Dnmt1-mediated maintenance methylation for proper function and survival of CNS neurons at multiple developmental stages. In mice with specific deletion of *Dnmt3a* in the CNS, global DNA methylation was unperturbed; however, mutant mice exhibited loss of hypoglossal motor neurons located in the brainstem, abnormal neuromuscular junctions, motor defects, and premature death [52]. This may result from changes in gene-specific DNA methylation and/or altered function of Dnmt3a-associated chromatin remodeling proteins that may cause misregulation of target genes that are critical for motor neuron survival and function. The phenotype of these mice was similar to transgenic mouse models of amyotrophic lateral sclerosis (ALS) [53–57], further implicating Dnmt3a in the pathogenesis of motor neuron degeneration. Animal models of Dnmt3b loss in the CNS have not been described to date; however, the identification of *DNMT3b* mutations in human mental retardation [58, 59] indicate the essential function of this protein for proper brain function which may include the preservation of neuronal integrity.

These effects of impaired Dnmt activity and resulting reduction in DNA methylation levels have been extended to primate models of neurodegeneration. In postmortem analyses of human brains, the promoter region of the Alzheimer’s disease (AD)-causing amyloid precursor protein (*APP*) gene was shown to be hypomethylated in older individuals, suggesting a link between enhanced transcriptional potential of a pathogenic gene and increased susceptibility to neurodegeneration with age [60]. Indeed, reduced DNA methyltransferase activity as well as decreased cortical levels of Dnmt1, Dnmt3a, and MeCP2 proteins were associated with increased expression of the AD-related genes *APP* and beta-site APP cleaving enzyme 1 (*BACE1*) in aged monkeys with AD-like neuropathology [61, 62]. Additional examination of brain tissues from AD patients revealed global DNA hypomethylation along with diminished immunoreactivity for DNMT1 and multiple components of the MeCP1/MBD2 methylation complex [63, 64], further supporting a neuroprotective role of DNA methylation in AD etiology.

Increased DNA methyltransferase levels and activity, as well as consequent hypermethylation of DNA are also associated with neuronal cell death. Elevated methyltransferase activity and DNA methylation were detected upon ischemic brain injury in mice, and either genetic or pharmacological inhibition of Dnmt1 was found to be neuroprotective in this mild stroke model [65]. Similarly, in a motor neuron degeneration model, Dnmt1 and Dnmt3a protein levels and total Dnmt enzyme activities were upregulated and mediated rapid increases in DNA methylation, while Dnmt inhibition protected neurons against apoptosis [66]. The increased Dnmt protein expression and DNA hypermethylation were paralleled in motor neurons of ALS patients [66]. Therefore, tight regulation of DNA methylation is also critical for the survival of neurons, as an imbalance to create either a hypo- or hypermethylated genomic state can cause neurodegeneration. The effects on transcription of particular genes is relevant to this finding, as repression of pro-survival genes and inappropriate activation of cell death genes could both lead to neuronal loss. Accordingly, altered methylation of genes that may contribute to ALS pathobiology was observed in human ALS brain-derived DNA, including both hypo- and hypermethylated genes [67]. Thus, disturbance of the steady state levels of DNA covalent modification, as also described above for histone acetylation and methylation, can contribute to altered chromatin remodeling activity that leads to neurodegenerative processes. This further emphasizes the importance of strict control of chromatin dynamics for cellular homeostasis and the physical and functional integrity of neurons.

While examples of dysregulated DNA methylation have not been documented for neurodegeneration affecting the retina, a recent study describes the expression of DNA methyltransferases in mouse retinal progenitors and mature neurons, highlighting distinct patterns of localization in different neuronal cell types that may reflect different developmental requirements for chromatin remodeling and/or differences in chromatin architecture that underlie cell-specific properties [68]. The role of specific Dnmts in mediating the function and maintenance of retinal neurons awaits further studies including cell type-specific ablation.

## ATP-dependent nucleosome remodelers mediate survival of specific cell types

ATP-dependent chromatin remodeling proteins function to non-covalently alter the structure of nucleosomes through modulation of DNA-histone interactions, thereby regulating chromatin compaction and DNA accessibility to proteins involved in diverse cellular processes such as DNA replication, recombination, repair, and transcription. The energy of ATP hydrolysis is utilized to disrupt nucleosomes by promoting histone sliding, repositioning, exchange, or eviction [4]. These nucleosome remodeling factors function as part of large multi-protein complexes and are recruited to target genes based on interactions with other subunits of these complexes that can recognize and bind to specific DNA sequences and/or histone and DNA modifications [69].

The importance of ATP-dependent chromatin remodeling proteins in neurodevelopment is demonstrated by the identification of mutations in the genes that encode these proteins, or proteins with which they interact, in human developmental disorders associated with intellectual disability [70–78]. A number of studies suggest that nucleosome remodelers also function to maintain cell viability, including neural tissues. The ATRX protein was one of the first chromatin remodeling factors associated with human genetic disease, with mutations in the gene causing α–thalassaemia mental retardation, X-linked (ATR-X) syndrome [71]. ATR-X syndrome patients exhibit a diverse range of clinical features including microcephaly and cognitive deficits indicating a critical role for ATRX in proper brain structure and function. Consistent with the human phenotype, conditional deletion of *Atrx* in the developing mouse forebrain resulted in reduced brain size and a significant loss of neuronal cells during corticogenesis [79, 80]. Hypocellularity was also detected at postnatal stages in the hippocampus, with mutant mice completely lacking a dentate gyrus. The neuronal loss occurred due to inappropriate apoptosis of neuroprogenitors and could be recapitulated in isolated neuroprogenitor primary cultures derived from the knockout mice, suggesting the involvement of cell autonomous cell death mechanisms [79]. In contrast, overexpression of *Atrx* in transgenic mice resulted in the generation of excessive neuroprogenitors [81], further supporting a role for Atrx in neuronal homeostasis.

In the mouse retina, loss of Atrx leads to a selective reduction in amacrine and horizontal cell populations in the postnatal period [82]. Various amacrine cell subtypes are affected, including glycinergic, cholinergic, and dopaminergic neurons [82]. Atrx is therefore important for the survival of inhibitory interneurons in both the retina and the brain, as loss of GABAergic interneurons is also observed in forebrain-specific knockout mice [80]. However, the survival of neuroprogenitors is unaffected in the *Atrx*-knockout retinas [82], implying differential spatial and temporal neuroprotective functions of Atrx. Interestingly, conditional deletion of *Atrx* in mouse testis causes prenatal apoptosis of proliferating Sertoli cells [83]. Physical interaction of Atrx with the testis-specific androgen receptor was shown to regulate transcription of tissue-specific target genes [83]. Thus Atrx-mediated maintenance of specific cell types in different tissues including the retina may result from the transcriptional regulation of tissue-specific Atrx targets that impinge on pro-survival pathways.

## Epigenetic mechanisms of neurotoxicity

While there is significant documentation of epigenetic factors associated with neuronal survival, there are few well-characterized mechanisms. Several potential triggers of neurotoxicity have been proposed to underlie degeneration of neurons in the retina and brain. These include oxidative stress [84], DNA damage [85, 86], mitochondrial dysfunction [87, 88], lack of neurotrophic support [89], and excitotoxicity [90]. Chromatin remodeling proteins have been implicated in mediating survival responses to some of these neurotoxic stimuli.

Oxidative stress results from the production of cytotoxic reactive oxygen species derived from cellular metabolism of oxygen. High metabolic activity, high proportions of polyunsaturated fatty acids that undergo lipid peroxidation, and exposure to visible light that induces photo-oxidation render the retina particularly susceptible to oxidative damage [91]. The inability of neurons to protect against or repair the damage caused by reactive oxygen species leads to their death. Bmi1 is a member of the Polycomb group family of chromatin modifiers, and its target genes include those involved in neuronal survival as well as antioxidant defenses [92]. Mice with Bmi1 deficiency exhibit retinal defects and increased neuronal death in the brain accompanied by increased apoptotic gene expression, reduced expression of antioxidant genes, and elevated levels of reactive oxygen species [92].

DNA damage is recognized as a significant mediator of neuronal cell death [85, 86]. Increases in both nuclear and mitochondrial DNA lesions have been reported for various neurodegenerative conditions including retinal degeneration [93–95]. Chromatin remodeling is a fundamental process in the protection of genomic integrity by DNA repair mechanisms [96], and thus it follows that chromatin remodeling proteins function in neuroprotective responses to DNA damage. Inhibition of HDAC1 was shown to result in double strand DNA breaks and neuronal death, while HDAC1 overexpression protected against DNA damage and neurotoxicity in cultured neurons [34]. In the *pcd* mouse model, Purkinje cell degeneration is associated with a large scale reorganization of chromatin and propagation of histone modifications that cause gene silencing (via trimethylated histone H4K20) and that signal DNA damage (phosphorylation of the histone variant H2AX) [42]. Defects in the DNA repair pathway and accumulation of DNA damage in these mice are presumed to trigger neuronal death.

Mitochondrial dysfunction is associated with various forms of retinal and brain degeneration, and mutation of mitochondrial genes can lead to neurodegenerative diseases [88, 97]. Mitochondria in mouse and human tissues were found to contain DNA methyltransferases and methylated mitochondrial DNA [66]. This study further showed that DNMT3a protein levels were upregulated in motor cortex mitochondria from ALS patients, suggesting that epigenetic regulation of mitochondrial genes may contribute to neurodegenerative phenotypes.

Neurotrophins are growth factors important for supporting neuronal function and survival and exert their neurotrophic effects through receptor-mediated intracellular signaling pathways. A number of these factors have demonstrated roles in neuroprotection of various retinal cell types in vitro and when administered to mouse models of retinal degeneration [89, 98]. Brain-derived neurotrophic factor (BDNF) has been reported to prevent retinal ganglion and amacrine cell death [99–101] and can also rescue photoreceptor loss in animal models of retinal degeneration [102, 103]. *BDNF* expression is controlled by a number of epigenetic factors [104] and can be modulated by DNA methylation and histone modification [105]. Neurotrophins such as BDNF in turn directly induce epigenetic changes to regulate the transcription of their target genes [106, 107]. Given the functional interaction between BDNF and MeCP2 [108] and the physical interaction of MeCP2 with Atrx [75], it is tempting to speculate that the amacrine cell loss observed in *Atrx*-knockout mouse retinas may result from misregulation of a common pathway involving all three factors that functions to promote retinal neuron survival.

Excitotoxicity is caused by the hyperactivity of glutamate receptors on the cell surface of neurons due to excessive levels of the excitatory neurotransmitter glutamate, which in turn produces multiple adverse effects that lead to neuronal cell death. Excitotoxic neuronal death is a common feature of acute and chronic neurodegenerative conditions affecting various regions of the CNS including the inner retina [90, 109]. Studies are emerging to implicate epigenetic mechanisms in excitotoxic processes. Glutamatergic inputs can induce chromatin remodeling in neurons, and this is inhibited by NMDA receptor antagonists [110]. Glutamate receptor expression is also subject to epigenetic regulation. The expression of glutamate receptor subunits is regulated by the transcriptional repressor REST/NRSF, which requires ATP-dependent chromatin remodeling mediated by Brg1 and histone deacetylation for gene silencing [111]. Increased REST expression was associated with suppression of the AMPA receptor subunit GluR2 and rescue of ischemia-induced neuronal cell death [112, 113]. Brg1-mediated recruitment of HDAC1 to the NMDA receptor subunit NR2B promoter leads to *NR2B* repression [114], while HDAC inhibition increases *NR2B* expression and NMDA receptor activity [115]. *NR2B* expression was also shown to be regulated by the histone methyltransferase Setdb1 [116], indicating multiple epigenetic levels of transcriptional control of glutamate receptors. In addition, the astroglial glutamate transporter EAAT2/GLT1 prevents glutamate-induced excitotoxicity, and its expression is regulated by DNA methylation in response to neuronal stimulation [117].

## Conclusions

Complementing the established role for epigenetic regulation in neuronal development and function are studies detailing the importance of chromatin remodeling for long-term survival. We have summarized the current knowledge of epigenetic strategies employed by neurons to withstand a lifetime of genetic and environmental insults in order to evade death. As these mechanisms continue to emerge, it is becoming clear that chromatin regulation of neuroprotection is complex and is impacted by a wide range of neurotoxic stimuli. Failure of these mechanisms leads to cellular dysfunction and neuronal loss, the key hallmarks of human neurodegenerative diseases.

The development of effective treatments for retinal degeneration and other neurodegenerative disorders requires a thorough understanding of the molecular events that control the survival of neurons and their demise in these diseases. The finding that changes in DNA and histone covalent modifications contribute to altered gene expression that leads to neuronal cell compromise can be exploited to generate therapeutic approaches targeted to chromatin modifying activities. For example, the association of reduced histone acetylation levels with neurodegenerative conditions has led to the extensive investigation of HDAC inhibitors as well as the proposal of HAT activators as therapeutics for neuronal degeneration [13, 118, 119]. However, the finely tuned epigenetic landscape of neurons, as evidenced by the interplay of different chromatin remodeling enzymes, poses significant challenges to the development of effective treatment regimes. Therapeutic efforts further need to address the differential temporal and spatial requirements for specific chromatin modifications or processing, as exemplified by the disparity of Atrx-mediated nucleosome remodeling in the embryonic forebrain and in the postnatal retina. It will be necessary to fully characterize the activities of individual chromatin modifying agents on specific genes in distinct neuronal cell populations, and in response to diverse neurotoxic triggers, to ensure specificity of targeted neuroprotective strategies and to avoid confounding off-target effects. With a heightened focus on investigation and discovery into the epigenetic programs controlling neuron survival and homeostasis in the retina and CNS, the promise of such directed treatments grows ever closer.
